# Supplementary material for: Lower limb joints’ contributions to ballet turnout during unipodal and bipodal jumps in fifth position in pre-professional dancers
Source: PeerJ. 2025 Oct 27;13:e20263. doi: 10.7717/peerj.20263 (PMC12574594; doi:10.7717/peerj.20263)
Supplement: Supplemental Information 1 [file peerj-13-20263-s001.docx]

**METHODS**

The range of motion (ROM) of each jump phase for each joint were also extracted from the time series. These variables were compared across phases and joints using repeated measures ANOVAs (p<0.05) and post hoc Newman–Keuls (Jamovi v. 2.5.3).

**RESULTS**Regarding the Assemblé (Table S1, Figure S1), the hip ROM was significantly greater during the preparation phase compared to the flight (post hoc: p < 0.001) and landing phases (post hoc: p < 0.001), with landing presenting the smallest ROM among all phases. At the knee, ROM significantly decreased from preparation to flight (post hoc: p < 0.001) and significantly increased from flight to landing (post hoc: p < 0.001), with the flight phase showing the lowest ROM compared to the others. Ankle ROM was significantly greater in the preparation phase compared to the other phases (post hoc: p < 0.001) and remained unchanged between flight and landing. Still in the Assemblé jump, during the preparation phase, knee ROM was significantly smaller than ankle ROM (post hoc: p < 0.001), while remaining similar to hip ROM (post hoc: p = 0.166). In the flight phase, knee ROM was significantly smaller compared to both hip and ankle (post hoc: p < 0.001), whereas hip and ankle ROM were similar to each other (post hoc: p = 0.885). In the landing phase, hip ROM was significantly smaller than both knee (post hoc: p < 0.001) and ankle (post hoc: p < 0.002) ROM.

**Table S1** - Descriptive statistics (mean ± standard deviation) and p-values for comparisons of hip, knee, and ankle joint range of motion (ROM) between jump phases and joints in *the Assemblé Dessus*.

|  | **Fase** | **Hip** | **Knee** | **Ankle** | **p ANOVA  (between joints)** | |
| --- | --- | --- | --- | --- | --- | --- |
|  | **Preparation** | 28 ± 10 | 23.3 ± 6.2 | 33.1 ± 6.8 | | <0.001 |
| **ROM**  **(degree)** | **Flight** | 19.9 ± 9.3 | 10.4 ± 5.4 | 20.7 ± 7.2 | | <0.001 |
|  | **Landing** | 12.7 ± 5.4 | 20.6 ± 7.3 | 18.5 ± 5.2 | | <0.001 |
| **p ANOVA**  **(between phases)** |  | <0.001 | <0.001 | <0.001 | |  |

**
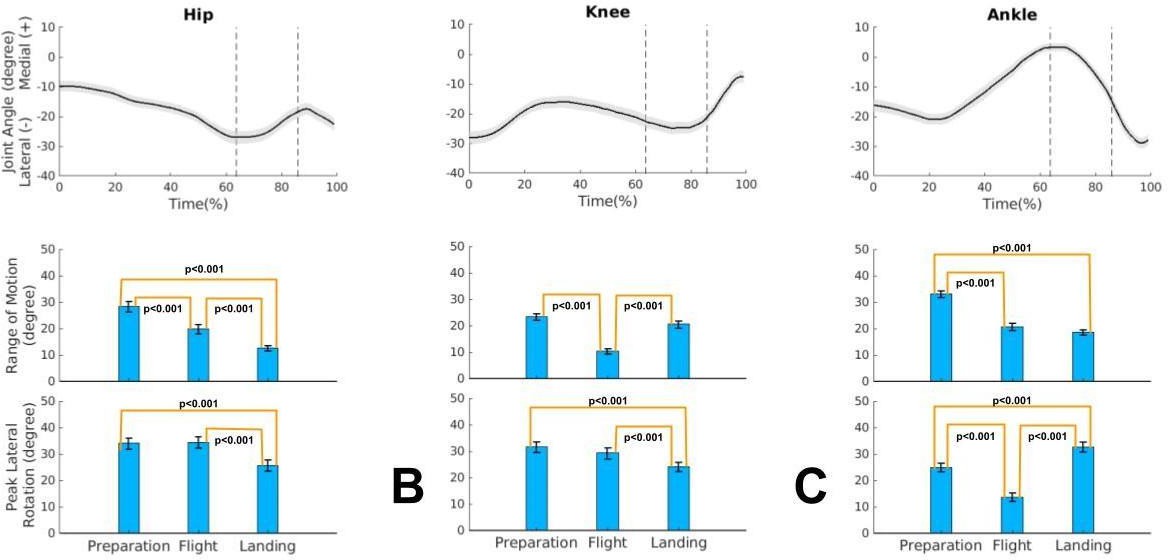
**

**A**

**Figure S1** – Upper panel: Ensemble mean and standard error of joint angles in the transverse plane for the hip (A), knee (B), and ankle (C) during the Assemblé Dessus. Lower panel: Mean and standard error of the range of motion and peak lateral rotation for the hip (A), knee (B), and ankle (C) across the three jump phases: preparation, flight, and landing.

Regarding the Sissone (Table 2, Figure 5), there was a significant increase in hip ROM from the preparation to the flight phase (post hoc: p < 0.002), followed by a decrease from flight to landing (post hoc: p < 0.001); compared to landing, the preparation phase showed a greater ROM (post hoc: p = 0.035). Knee ROM was significantly greater during preparation compared to the other phases (post hoc: p < 0.001), and the smallest amplitude occurred during flight in comparison to the other phases (post hoc: p < 0.001). Ankle ROM significantly decreased from preparation to flight (post hoc: p < 0.001) and increased from flight to landing (post hoc: p < 0.001), remaining similar between the preparation and landing phases.When comparing joints within each phase, knee ROM was significantly greater than the other joints during the preparation phase of the Sissone (post hoc: p < 0.001). In the flight phase, hip ROM was significantly greater than that of the knee and ankle (post hoc: p < 0.001), while in the landing phase, hip ROM was significantly smaller compared to the other joints (post hoc: p < 0.001).

**Table S2** - Descriptive statistics (mean ± standard deviation) and p-values for comparisons of hip, knee, and ankle joint range of motion (ROM) between jump phases and joints in the *Sissone Ouvert.*

|  | **Fase** | **Hip** | **Knee** | **Ankle** | **p ANOVA  (between joints)** | |
| --- | --- | --- | --- | --- | --- | --- |
|  | **Preparation** | 16.3 ± 4.7 | 40± 12 | 21.2 ± 7.7 | | <0.001 |
| **ROM**  **(degree)** | **Flight** | 21.4 ± 8.4 | 14.2 ± 4.5 | 13.3 ± 3.8 | | <0.001 |
|  | **Landing** | 13.0 ± 4.1 | 20.0 ± 6.1 | 18.8 ± 6.0 | | <0.001 |
| **p ANOVA**  **(between phases)** |  | <0.001 | <0.001 | <0.001 | |  |


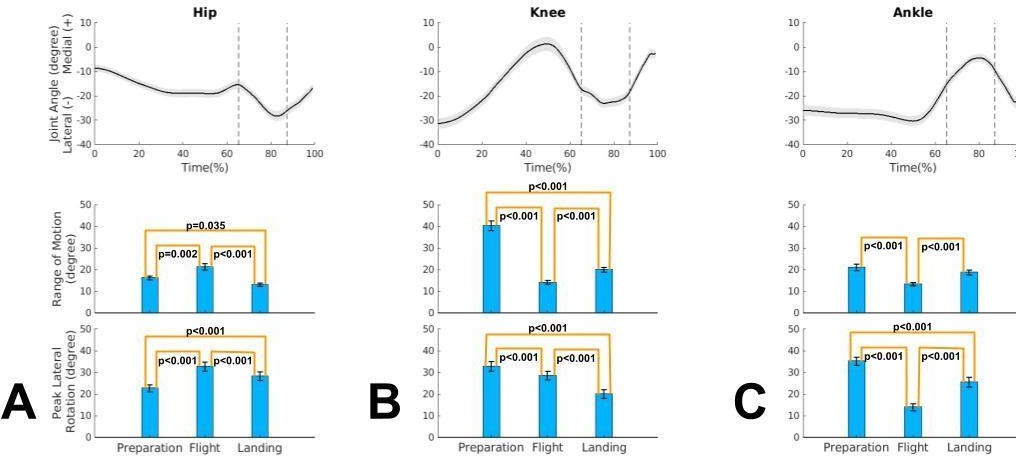


**Figure S2** – Upper panel: Ensemble mean and standard error of joint angles in the transverse plane for the hip (A), knee (B), and ankle (C) during the *Sissone Ouvert*. Lower panel: Mean and standard error of the range of motion and peak lateral rotation for the hip (A), knee (B), and ankle (C) across the three jump phases: preparation, flight, and landing.
